# Individual differences in attention influence perceptual decision making

**DOI:** 10.3389/fpsyg.2015.00018

**Published:** 2015-02-05

**Authors:** Michael D. Nunez, Ramesh Srinivasan, Joachim Vandekerckhove

**Affiliations:** ^1^Department of Cognitive Sciences, University of California, IrvineIrvine, CA, USA; ^2^Department of Biomedical Engineering, University of California, IrvineIrvine, CA, USA; ^3^Institute for Mathematical Behavioral Sciences, University of California, IrvineIrvine, CA, USA

**Keywords:** electroencephalography (EEG), steady-state visual evoked potential (SSVEP), Phase-locking, hierarchical Bayesian modeling, diffusion models, individual differences, perceptual decision making

## Abstract

Sequential sampling decision-making models have been successful in accounting for reaction time (RT) and accuracy data in two-alternative forced choice tasks. These models have been used to describe the behavior of populations of participants, and explanatory structures have been proposed to account for between individual variability in model parameters. In this study we show that individual differences in behavior from a novel perceptual decision making task can be attributed to (1) differences in evidence accumulation rates, (2) differences in variability of evidence accumulation within trials, and (3) differences in non-decision times across individuals. Using electroencephalography (EEG), we demonstrate that these differences in cognitive variables, in turn, can be explained by attentional differences as measured by phase-locking of steady-state visual evoked potential (SSVEP) responses to the signal and noise components of the visual stimulus. Parameters of a cognitive model (a diffusion model) were obtained from accuracy and RT distributions and related to phase-locking indices (PLIs) of SSVEPs with a single step in a hierarchical Bayesian framework. Participants who were able to suppress the SSVEP response to visual noise in high frequency bands were able to accumulate correct evidence faster and had shorter non-decision times (preprocessing or motor response times), leading to more accurate responses and faster response times. We show that the combination of cognitive modeling and neural data in a hierarchical Bayesian framework relates physiological processes to the cognitive processes of participants, and that a model with a new (out-of-sample) participant's neural data can predict that participant's behavior more accurately than models without physiological data.

## 1. Introduction

The joint analysis of physiological and behavioral data has been a topic of recent interest. In a string of publications, a number of research groups (Forstmann et al., 2010; Turner et al., 2013; Cassey et al., 2014) have presented work in which neurophysiological data are linked to parameters of cognitive or behavioral process models (see also Palmeri et al., in preparation). The goal of these modeling exercises is not only to evaluate the predictive power of brain activity for behavior, but also to elucidate the nature of this prediction. The use of cognitive models with neural data and cognitive parameters permits more psychologically interpretable labeling of the neurophysiological measurements, providing links between brain activity, cognition, and behavior.

In the present paper, we apply a cognitive model constrained by EEG data to fit accuracy and response times of multiple individuals from a perceptual decision making task. The goal of the model fit is twofold: (1) to demonstrate the superior generalizability of such a model as compared to model variants without neural input components and (2) to evaluate the hypothesis that individual differences in enhancement or suppression of visual attention, as measured by EEG, contribute to individual differences in cognition and thus to individual differences in accuracy and/or reaction time in the task.

In order to show out-of-sample generalizability, we first fit the model to a training set of participants and obtain the requisite (population-level) linking parameters, and then make predictions about the behavior of a new participant to which the model was not trained. In the sections that follow, we will describe (1) the cognitive process model that we have chosen, (2) the task to which it is applied and the EEG data that we collected, (3) a series of three models of increasing complexity, of which the model with external attentional EEG covariates is the most complex, (4) the results of the generalization exercise and (5) evaluation of the hypothesis.

### 1.1. Steady-state visual evoked potentials as a measure of attention

In this study, we will demonstrate how attentional mechanisms can explain individual differences in perceptual decision making as estimated by a cognitive model. In a typical visual attention experiment, the signal stimulus is attended and preferentially processed while competing stimuli (i.e., visual noise) are not further processed. A number of studies have demonstrated that a measure of the deployment of attention can be obtained by using flickering stimuli and electroencephalographic (EEG) recordings of the (frequency tagged) steady-state visual evoked potentials (SSVEPs) (Morgan et al., 1996; Müller et al., 1998; Ding et al., 2006; Bridwell and Srinivasan, 2012; Garcia et al., 2013). SSVEPs are narrow band responses at the visual flicker frequencies and flicker harmonics of a stimulus (Regan, 1977). When a stimulus is attended, the SSVEP is enhanced, and when a stimulus is not attended or suppressed, the SSVEP is diminished. This approach has been used to investigate individual differences in attention strategy in detection and discrimination tasks. Bridwell et al. (2013) found that only a subset of participants could deploy the optimal attention strategy and modify their strategy by the task demands. An SSVEP approach has also been used to show that individuals are trained by their own experiences. Individuals with attentional training due to a history of fast-action video gaming have been found to preferentially suppress noise rather than enhance the signal, and those individuals performed better at vigilance tasks (Krishnan et al., 2013).

### 1.2. Diffusion models for two-choice response times

**Figure 1:**
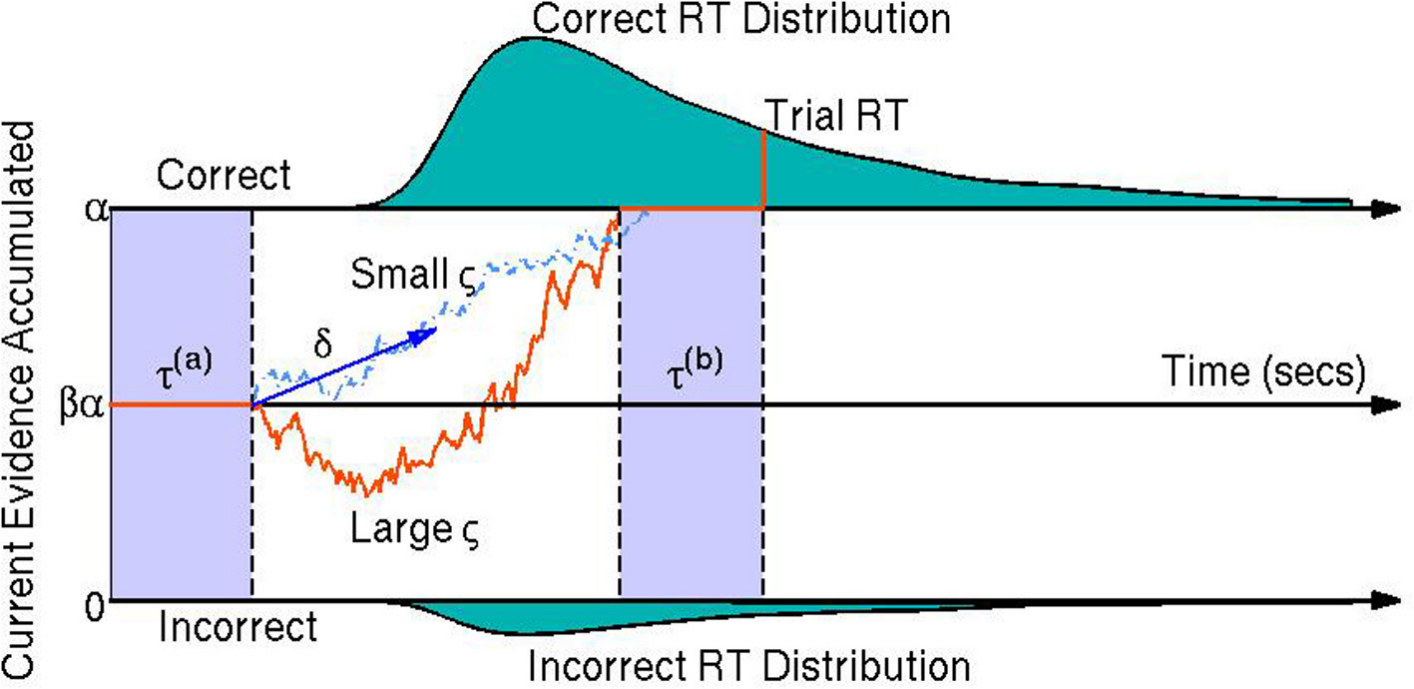
**A visual representation of the diffusion model**. The orange line represents the participant's stochastic evidence accumulation process during one trial. When a participant accumulates enough evidence over time for a correct or incorrect response (graphically represented by the top and bottom boundaries at 0 and α, respectively) a decision is made. The drift rate δ is the mean rate of evidence accumulation (evidence units per second) during the participant's decision time on one trial. The bias parameter β represents a bias of the participant toward one choice or the other (set to $\frac{1}{2}$ when the model parameters are expressed in terms of correct over incorrect evidence instead of choice A over choice B evidence). The non-decision time τ is the portion of the participant's reaction time (RT) during the trial not associated with decision making, equal to the sum of encoding/preprocessing time τ^(*a*)^ and motor response time τ^(*b*)^ which are not estimable. The boundary separation parameter α represents the amount of relative evidence needed to make a decision. Another parameter is the variability in the evidence accumulation process, the diffusion coefficient ς. In this trial the diffusion coefficient is large in comparison to a smaller diffusion coefficient as shown by the light blue dashed line. The teal shaded areas represent the correct (top) and incorrect (bottom) reaction time distributions. In this example the systematic component of the decision making process is positive δ > 0 indicating a mean trend toward correct responses. However, incorrect responses can still be reached due to the random component of the decision making process (the diffusion coefficient ς). Larger ς indicate that a participant would be increasing likely to make faster decisions, but have closer to chance performance (i.e., an accuracy of β).

Diffusion models are a class of sequential-sampling models for reaction time (RT) and response data that can capture the joint distribution of RT and accuracy in speeded choice tasks. This family of models has been useful in explaining between- and within-participant variability in two-alternative forced choice decision making experiments (Vandekerckhove et al., 2008, 2011). Diffusion models also add to the analyses of participants' behavior by assuming underlying cognitive processes which have some empirical validation (Voss et al., 2004). In particular, they assume that at each trial, participants obtain relative evidence from a stimulus over time until sufficient evidence is accumulated to exceed the threshold for one of the two choices (Stone, 1960; Link and Heath, 1975; Ratcliff, 1978). This process of relative evidence accumulation is modeled as a Wiener diffusion process (or *Brownian motion*) and can be thought of as a continuous random walk process—that is, a random walk process where in each infinitesimal time step, the evidence increases by a random amount according to a normal distribution with some mean and some instantaneous variance (Ratcliff, 1978). A visual representation of the model is provided in Figure 1.

Fitting RT and choice behavior using the diffusion model is a useful behavioral analysis tool since the model's parameters have interpretable psychological correlates. The drift rate δ_*j*_ represents the mean rate of evidence accumulation of participant *j* during their decision process. The drift rate is thought to reflect the quality of evidence the participant obtains during an experimental trial (Ratcliff et al., 2001). The diffusion coefficient ς_*j*_ is the parameter that represents the amount of variability in the evidence accumulation process within one trial (i.e., the instantaneous variance). The bias parameter β_*j*_ is the proportion of bias a participant *j* has in favor of choice A over choice B (it should be noted that we fix the bias parameter to $\frac{1}{2}$ in this paper since we model behavior as correct vs. incorrect trials instead of choice A over choice B trials). The non-decision time τ_*j*_ is the amount of time during the response process that is not associated with the decision making process, such as preprocessing of the stimulus and/or motor response time. Finally, the boundary separation parameter α_*j*_ represents the amount of relative evidence needed to make a decision and is typically manipulated by task instructions emphasizing either speed or accuracy (Ratcliff et al., 2001; Voss et al., 2004). It is important to note that the model is not identifiable unless we constrain at least one of the parameters that pertain to the evidence dimension (i.e., diffusion coefficient ς, drift rate δ, or boundary separation α).

### 1.3. The case for hierarchical bayesian models

Recent advances in mathematical psychology have introduced hierarchical Bayesian versions of cognitive models (Rouder et al., 2005; Vandekerckhove et al., 2011). The advantages of these hybrid modeling–measurement strategies include more principled (Bayesian) statistical inference, increased statistical power (Vandekerckhove et al., 2010), and interpretability of results in terms of psychological concepts rather than statistical summary (Vandekerckhove, 2014). The use of cognitive models as measurement tools has become known as *cognitive psychometrics* (e.g., Batchelder, 2010).

The hierarchical Bayesian process modeling framework is ideally suited for the joint analysis of multiple modes of data—(Turner et al., 2013) describe three such joint modeling strategies and (Vandekerckhove, 2014) describes a fourth. One strategy afforded by hierarchical Bayesian models involves constraining the estimation of cognitive process models by introducing the brain data as (fixed) covariate information. This strategy carries the disadvantage that it does not by default allow for measurement variance on the neurophysiological side, but has the advantage of being relatively straightforward to implement in a computationally efficient fashion. By conditioning the estimation of the cognitive parameters on brain data (or other external covariates), it is expected that unexplained variability between participants can be reduced, and consequently that such a model should perform better in generalization tests.

Interindividual variability (i.e., variability in the participant-level cognitive parameters; changes over subscript *j*) in diffusion models has been previously analyzed by fitting a diffusion model to each participant individually then comparing parameters across model fits. The individual differences were then gauged by statistical analyses on the models' resulting maximum likelihood parameter estimates (Ratcliff et al., 2001; Wagenmakers et al., 2008). Some limitations to this technique are that large sample sizes are needed for diffusion model parameter estimation, that shared condition-level differences across individuals cannot be easily evaluated (Wagenmakers, 2009; Vandekerckhove et al., 2011), and that statistical uncertainty is not propagated across stages of the analysis. Hierarchical Bayesian methods along with Monte Carlo sampling techniques allow for the estimation of complex models. These methods have been used to explain individual differences in the diffusion model and other cognitive models without the need for large sample sizes (Lee, 2008; Lee and Newell, 2011; Vandekerckhove et al., 2011). Additionally, the hierarchical framework allows for between-participant variability to be explained when each participant's diffusion model parameters are functionally related to known exogenous data (e.g., physiological data).

### 1.4. Constraining model parameters with EEG data

We assume that brain activity compels cognition, which in turn drives participant behavior. Assuming attention constrains one or more of the cognitive processes in perceptual decision making, then as a consequence of attentional mechanisms we expect SSVEPs to help explain between-participant variability in the parameters of the diffusion model and thus between-participant variability in RT and accuracy. In one study, an occipital SSVEP amplitude was shown to track visual sensory evidence over the time course of a trial, suggesting that SSVEPs can reflect the evidence accumulation process itself (O'Connell et al., 2012). The experimental stimulus used in this study involves a flickering signal overlayed on time-varying visual noise, designed to evoke separate SSVEP responses to the signal and the visual noise, which we expect will explain individual differences in the model parameters and behavior.

We hypothesize increased within-trial evidence accumulation rates, reflected by increased drift rates, for those subjects who suppressed attention to the visual noise. We further hypothesize that another benefit of attention for RT and accuracy is a result of reduced within-trial variability in the accumulation of evidence. Thus, we predict an across-individuals relationship between enhanced attention to the signal and decreased diffusion coefficients.

As mentioned above, one of the parameters of the diffusion model must be fixed rather than estimated (either diffusion coefficient ς, drift rate δ, or boundary separation α). For the present study a variable boundary separation across conditions is not a valid interpretation of the data since the changes between conditions occur unannounced, leaving the participant with no opportunity to adapt strategies (e.g., switch between a speed or accuracy strategy) in response to stimulus changes. In our parameterization, we leave the diffusion coefficient ς free to vary, set α to one evidence unit, and assume no bias (β = $\frac{1}{2}$) toward correct responses. The joint density *f* of RT *t* and accuracy *w* of this simplified diffusion model is given in Equation 1. The density is derived from the limiting approximation given by Ratcliff (1978) where *z* = $\frac{1}{2}$α and α = 1.

(1) $$\{\begin{array}{l} f(t,\;w=0\;|\;\varsigma^{2},\;\tau ,\;\delta )=\pi \varsigma^{2}e^{-\frac{1}{2}\left[\frac{\delta }{\varsigma }+\delta^{2}\left(t-\tau \right)\right]}\\ \begin{array}{c} \;\sum_{k=1}^{+\infty }\left[k\;\sin\left(\frac{1}{2}\pi k\right)e^{-\frac{1}{2}k^{2}\pi^{2}\varsigma^{2}\left(t-\tau \right)}\right] \end{array}\\ f(t,\;w=1\;|\;\varsigma^{2},\;\tau ,\;\delta )=f(t,\;w\;=\;0\;|\;\varsigma^{2},\;\tau ,\;-\delta ) \end{array}$$

In what follows, we will use the effect of attention, as measured by SSVEPs, to constrain diffusion model parameter estimates (in our case δ_*j*_, ς_*j*_, and τ_*j*_). In particular, we assume that, on each trial, a participant's attention is reflected in phase locking (i.e., SSVEPs) to the attended visual signal and decreased phase locking to the unattended visual noise.

We will demonstrate that the hierarchical Bayesian SSVEP-driven diffusion model has predictive ability as well as descriptive ability—more specifically, that our ability to predict each participant's accuracy and RT behavior is improved by including the SSVEP measures of attention processes.

## 2. Materials and methods

### 2.1. Participants

The following study was approved by the University of California, Irvine Institutional Review Board and was performed in accordance with APA standards. Informed consent was obtained from each of the seventeen participants (8 females and 9 males) who took part in the study. The mean age of 16 of the participants was 25 with an age range of 21–30. Another participant was over 45 years of age. Sixteen participants self-identified as being right handed while another identified as being left or ambidextrous. All participants had at least 20/30 vision or corrected vision as measured by a visual acuity chart available on the internet (Olitsky et al., 2013). No participants reported any history of neurological disorder. Each participant completed the experiment in one session within 2.5 h.

### 2.2. Experimental stimulus

The participants were given a two-alternative forced-choice perceptual decision making task in which they were asked to differentiate the mean rotation of bars within a circular field of bars that deviated randomly from mean rotation. One half of the trials had a mean bar rotation of 45° while the other half had a mean rotation of 135°. The bar field was flickered against a time-varying noise pattern.

**Figure 2:**
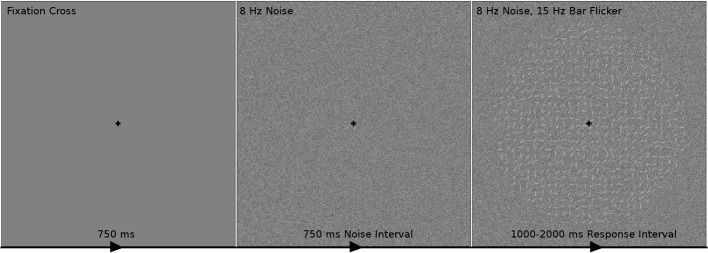
**The time course of one trial of the experimental stimulus**. The participant first fixated on a black cross for 750 ms indicating the beginning of a trial. The participant then observed visual contrast noise changing at 8 Hz for 750 ms while maintaining fixation. A circular field of small oriented bars flickering at 15 Hz overlaid on the changing visual noise was then shown to the participant for 1000–2000 ms. The task was to indicate during this response interval whether the bars were on average oriented toward the “top-right” (45° from the horizontal line; as in this example) or “top-left” (135°) corners. It was assumed that the participant's decision making process began at the start of the response interval. After the response interval, the fixation cross was shown in isolation for 250 ms to alert the participant that the trial was over and to collect remaining responses.

The participants viewed each trial of the experimental stimulus on a monitor in a dark room. The time course of one trial is shown in Figure 2. Participants were positioned such that the entire circular field of small oriented bars had a visual angle of 9.5°. Within each trial the participant first saw a black cross for 750 ms in the middle of the screen on which they were instructed to maintain fixation throughout the trial. The participant then observed visual contrast noise changing at 8 Hz for 750 ms; this time period of the trial will be referred to later in this paper as the *noise interval*. The participant then observed a circular field of small oriented bars flickering at 15 Hz overlaid on the square field of visual noise pattern changing at 8 Hz and responded during this time frame, henceforth referred to as the *response interval*. The visual noise and bar field are modulated at constant rates (8 and 15 Hz, respectively) to evoke frequency-tagged signal and noise responses in the cortex which we measured as steady-state visual evoked potentials (SSVEPs). The SSVEP responses at the signal frequencies (15 Hz and its harmonics) and at the contrast noise frequencies (8 Hz and its harmonics) were used to measure the effect of attention to the signal stimulus and noise stimulus. The display time of the response interval was sampled between 1000 and 2000 ms from a uniform distribution. After this display period the black fixation cross was shown in isolation for 250 ms to alert the participant the trial was over and to collect any delayed responses.

Three levels of variance of bar rotation and three levels of contrast noise were used to modulate the task difficulty. In the first level of bar rotation variance, each bar was drawn from a uniform 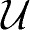
(−30°, 30°) distribution centered on the mean angle. In the two other levels, the rotations of each bar were drawn from 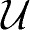
(−35°, 35°) and 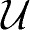
(−40°, 40°), respectively. The three levels of contrast noise were 30% contrast noise, 45% contrast noise and 60% contrast noise. The 30% contrast noise condition was obtained by the addition of a random draw from a 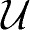
(−15%, 15%) distribution to the luminance of each pixel in a square field. Baseline luminance was 50%. The other contrast noise conditions were obtained similarly. Each participant was shown 90 trials from each bar rotation-noise condition combination.

The bar rotation (BR) variance manipulation was hypothesized to modulate each participant's diffusion coefficient since the participant would have more variable information in harder trials. Considering each bar's rotation as a unit of information contributing to a “left” or “right” response, information would be more variable in trials that sampled the BRs from wider uniform distributions. It was thought that contrast noise would degrade the amount of information each bar gave to the decision process thus leading to smaller drift rates in trials with higher noise contrast.

### 2.3. Behavior and EEG collection

Participants first completed a training session of 36 trials each. Participants were asked to complete a second training set if their percentage accuracy was subjectively judged by the experimenter to not converge to a stable value. Each participant completed 6 blocks of 90 trials each for a total of 540 trials with breaks between each block of variable time. Each trial lasted randomly (uniformly) from 2.75–3.75 s. Participants were asked to respond during the 1–2 s response interval as accurately as possible, with no-answer trials considered as incorrect. To maintain participant performance, auditory feedback was given after the response interval to the alert the participant if they were correct or incorrect. Performance feedback was also provided between blocks by displaying on the screen the percentage of trials answered correctly in that block. The behavioral data consists of each participant's accuracy and RT during each trial.

High-density electroencephalography (EEG) was collected using Electrical Geodesics, Inc.'s 128-channel Geodesic Sensor Net and Advanced Neuro Technology's amplifier with electrodes sitting on the participant's scalp throughout the duration of the experiment. Electrical activity from the scalp was recorded at a sampling rate of 1024 samples per second with an online average reference using Advanced Neuro Technology's digitization software. The EEG data was then imported into MATLAB for offline analysis.

Linear trends were removed from the EEG data. As we were only interested in 1–50 Hz EEG, the following filters were applied to each channel: (1) A high pass Butterworth filter with a 1 Hz pass band with 1 dB ripple and 0.25 Hz stop band with 10 dB attenuation, (2) a stopband Butterworth filter with 59 and 61 Hz pass bands with 1 dB ripple and 59.9–60.1 Hz stop band with 10 dB attenuation (to remove power-line noise), and (3) a low pass Butterworth filter with a 50 Hz pass band with 1 dB ripple and 60 Hz stop band with 10 dB attenuation. Artifactual data thought to be generated by phenomena outside of the cortex were removed from the EEG data using a paradigm involving Independent Component Analysis (ICA): First, any trials or channels were rejected that had time-courses unusual for cortical activity and/or had properties that ICA is deemed to not extract well, such as trials with high frequency activity indicative of muscle activity, trials or channels with high 60 Hz amplitude indicative of power-line noise suggesting poor electrode-to-skin connection, or trials with sudden high amplitude peaks that cannot be generated by cortical activity (Delorme et al., 2007). Second, ICA was used to remove linear mixtures of channel time-courses that did not subjectively correspond to EEG data in spatial map on the scalp, in power spectrum, and/or in event-related potential (ERP). Typical artifactual components include: those components with spatial maps of highly weighted electrodes near the eyes suggestive of eye movements, those components with high amplitudes at high frequencies and low amplitudes at low frequencies suggestive of muscle activity, and spatial maps of highly weighted singular electrodes suggestive of poor electrode-scalp connectivity. A final cleaning step was performed by rejecting any trials that had high amplitudes not typical of cortical electrical activity.

For each participant, steady-state visual evoked potentials (SSVEPs) to the visual noise and signal (the circular bar field) were found at each electrode. In this experiment a steady-state response was defined by the consistency in phase at the frequencies of the stimulus (8 and 15 Hz) and the harmonic frequencies of the stimulus (16, 24, 32, 40, 48, 30, and 45 Hz). The uniformity of phase across trials was measured by the Phase Locking Index (PLI) across trials. The PLI is a statistical characterization of phase synchronization resulting from an experimental stimulus and has been shown to be successful in characterizing cortical signals (Rosenblum et al., 1996; Sazonov et al., 2009). The PLI ignores signal amplitude and ranges from 0 (all trials out-of-phase) to 1 (all trials in-phase; Tallon-Baudry et al., 1996). The equation used for PLI is provided in Equation 2. PLI is the average of ≈ 540 trials of amplitude normalized Fourier coefficients of the time interval. For each electrode *e* and participant *j*, PLI is defined as a function of frequency *f*.

(2) $${\text{PLI}}_{ej}(f)=\;\left|\frac{1}{540}\sum_{i=1}^{540}\frac{F_{iej}(f)}{\left|F_{iej}(f)\right|}\right|$$

**Figure 3:**
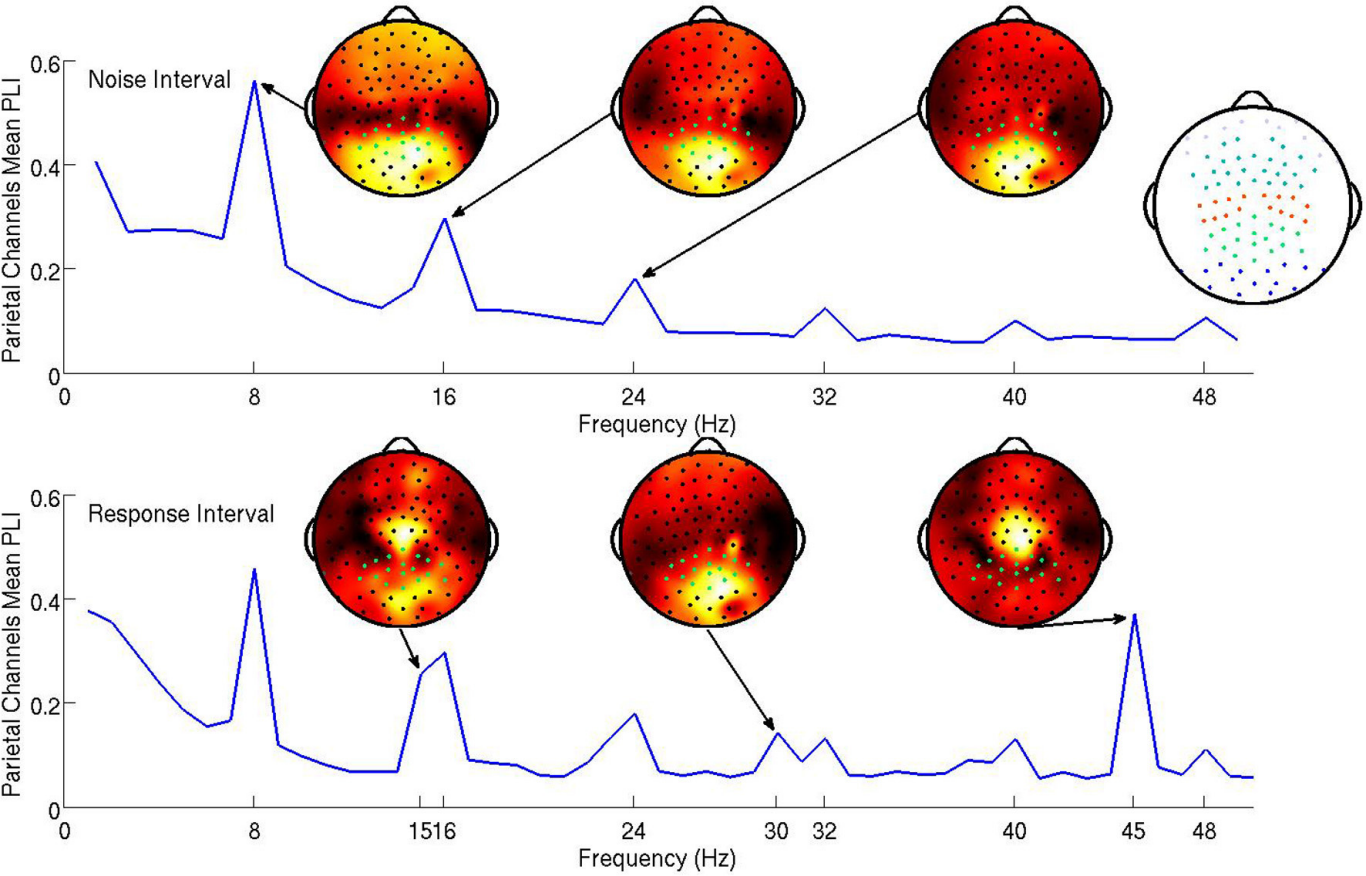
**The subject mean parietal channel PLI at all frequencies in the noise interval (top panel) and the response interval (bottom)**. The resolution of the the PLI spectra in the top plot is approximately 1.3 Hz due to the PLI being a function of Fourier transforms of 750 ms epochs. The resolution of the PLI spectra in the bottom plot is 1 Hz as the Fourier transforms are of 1000 ms epochs. The 15 and 16 steady-state responses during the response interval are separable when using 1000 ms epochs. Also shown are subject mean PLI topographies (at 8, 16, and 24 Hz during the noise interval and 15, 30, and 45 Hz during the response interval, each on a standardized scale) indicating where the maximum subject mean PLI is located on the scalp in relation to the parietal electrodes (highlighted green). It is clear from these topographies that using only parietal electrodes will not capture all of the steady-state response information. An index of electrode locations is also provided in the top right. Prefrontal, frontal, central, parietal, and occipital electrode groups are colored light blue, teal, orange, green, and blue, respectively.

The steady-state responses to the visual noise were analyzed based on both the 750 ms noise interval and the first 1000 ms of the response interval while the steady-state responses to the signal were analyzed based only on the first 1000 ms of the response interval. Because steady-state responses located in parietal electrodes have been successfully related to attentional mechanisms in past studies (Ding et al., 2006; Bridwell and Srinivasan, 2012), electrical activity at parietal electrodes was hypothesized to be most descriptive of cognitive processes in the visual decision making task. The subject mean PLI at all frequencies averaged over parietal channels is shown in Figure 3. Topographic maps of the distribution of the PLI are shown at the fundamental and first two harmonics for signal and noise frequencies. It is clear that the SSVEP is broadly distributed over frontal, parietal, and occipital networks, as has been found in other studies (Ding et al., 2006; Bridwell and Srinivasan, 2012; Krishnan et al., 2013). The mean PLIs over prefrontal, frontal, central, parietal, and occipital electrode groups for each of the evoked frequencies were used as predictors in the model.

We expect the evoked cortical networks to change dependent upon the flicker frequencies of the stimulus (Ding et al., 2006; Bridwell and Srinivasan, 2012), as shown by the stimulus response in Figure 3 where the spatial distributions of the fundamental and harmonic responses are quite different. However, we do not expect the behavior of these harmonics to be uncorrelated. To avoid multicollinearity, we performed two principal components analyses (PCAs; on the noise and signal frequencies separately) to obtain a smaller number of PLI measures from uncorrelated cortical networks. The first PCA reduced 60 PLI variables (5 cortical locations by 6 noise harmonics in both the noise and response intervals) to 16 principal components. The second PCA transformed 15 PLI variables (5 cortical locations by 3 signal harmonics) to 15 principal components. Our criteria for which principal components to include in the hierarchical Bayesian models were (1) based upon the improvement of in-sample predictive power as we increased the number of principal components, resulting in candidate principal components and (2) then based upon the out-of-sample predictive power of the candidate principal components.

### 2.4. Hierarchical Bayesian models

All trials from every participant were used for model fitting except those trials in which there was deemed to be EEG artifact and those trials during which the participant made no response or responded more than once. Since our models do not account for non-decision making trials, exceedingly fast trials (faster than 250 ms) were excluded as well.

The marginal likelihood for the model—that is, the predicted distribution of the data conditional on all parameters—is the first passage time distribution of a Wiener process with constant drift. We call this probability density function the *Wiener distribution*. For each trial *i*, subject *j*, and condition *k*, the observed accuracy *w*_*ijk*_ and RT *t*_*ijk*_ were combined in a two-element vector **y**_*ijk*_. These values were then assumed to be drawn from a joint distribution:

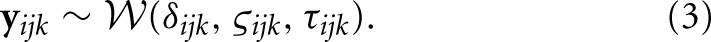



We applied a sequence of three models—each adding a new feature—to the data.

### 2.5. Model 1: no individual differences

**Figure 4:**
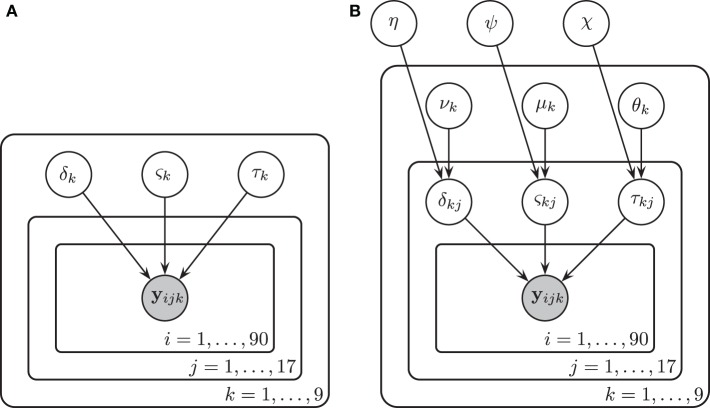
**A graphical representation of **Model 1** (A) and **Model 2** (B)**. In **Model 1**, drift rates δ_*k*_, diffusion coefficients ς_*k*_, and non-decision times τ_*k*_ were assumed to vary over conditions *k* but remain invariant across participants *j* and trials *i*. There were three bar rotation conditions and three contrast noise conditions. Here *k* denotes each bar rotation and contrast noise pair. In **Model 2**, drift rates δ_*jk*_, diffusion coefficients ς_*jk*_, and non-decision times τ_*jk*_ were assumed to vary over both conditions and participants. Each of these parameters are in turn assumed to be drawn from normal distributions with means that varied over conditions *k* and with variances that did not vary across conditions.

We assumed in **Model 1** that all three diffusion model parameters were constant across participants (i.e., that all participants were *identical*), and depended only on the experimental condition *k*. The diffusion model was fit to the RT and accuracy data of all 17 participants under the assumption that all participants had the same drift rate δ_*k*_, diffusion coefficient ς_*k*_, and non-decision time τ_*k*_ that were variable across condition *k* but not variable across participant *j*. Here *k* denotes both the particular BR condition and the particular contrast noise condition-level, *k* = 1, …, 9. A graphical representation of **Model 1** is provided in Figure 4A.

The assumptions of the model, together with the prior distributions for the parameters, appear below. The priors for the drift rate δ_*k*_ and non-decision time τ_*k*_ were truncated normal distributions due to the knowledge of the natural constraints of the diffusion model and prior knowledge of acceptable values for similar tasks. Note that the second parameter of the normal distributions below represent the variance.

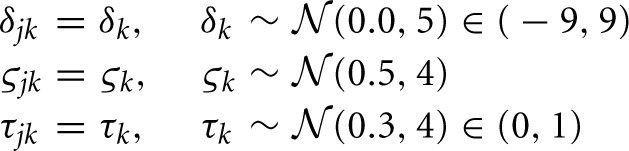



### 2.6. Model 2: individual differences

In **Model 2** we assumed that participants differ but are draws from a single superordinate population (i.e., participants are *exchangeable*). Consequently, the drift rate δ_*jk*_, diffusion coefficient ς_*jk*_, and non-decision time τ_*jk*_ varied by both subject *j* and condition *k*. Subject-level parameters were assumed to be drawn from normal distributions with means that were variable over condition only. Variances were assumed to be invariant across conditions to maintain model simplicity (i.e., the model assumes *homoscedasticity* in the parameters). The prior distributions of the parameters are listed below.

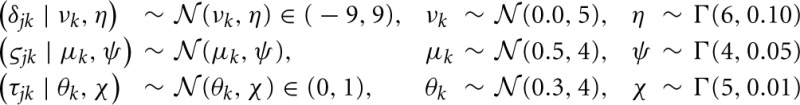



A graphical representation of **Model 2** is provided in Figure 4B.

### 2.7. Model 3: individual differences with neural correlates

With **Model 3**, we will attempt to explain any individual differences in cognitive parameters by introducing the neural data as explanatory variables. The model is similar to **Model 2**, but additionally includes a regression structure to explain variability in subject-level model parameters with steady-state PLI values.

In order to avoid multicollinearity, PLIs were first subjected to a principal component analysis (PCA), and the resultant independent components were used as predictors. The PCA was performed on the noise and signal frequencies separately. The first PCA reduced 60 PLI variables to 16 principal components and the second PCA transformed 15 PLI variables into 15 components. The criterion used to determine which principal components to include was the out-of-sample predictive power of each model. Predictive power was measured as *R*^2^_pred_, a measure of the percentage of total between-subject variance explained, in this case of the correct-RT medians of each condition. The equation used for *R*^2^_pred_ is provided in the Supplemental Materials.

**Figure 5:**
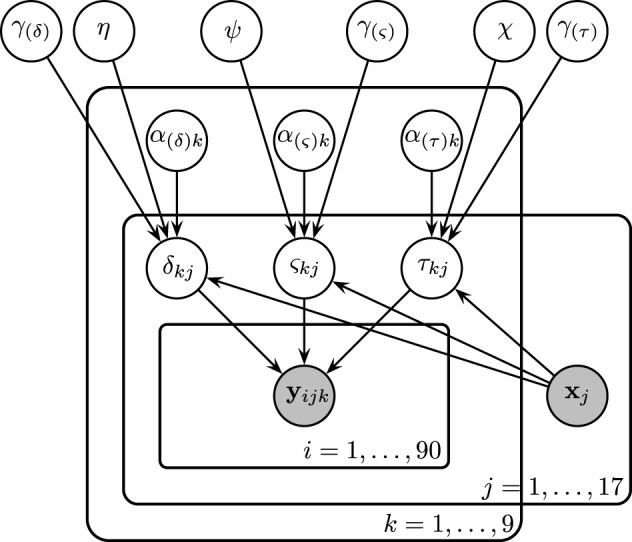
**Graphical representation of **Model 3****. Drift rates δ_*jk*_, diffusion coefficients ς_*jk*_, and non-decision times τ_*jk*_ were assumed to vary over both conditions and participants. Each of these parameters are assumed to be drawn from normal distributions with means of the form α_*k*_ + ***x***^**⊺**^_*j*_**γ**, where ***x***_*j*_ is the vector of SSVEP responses of subject *j*, and with variances that did not vary across conditions. As an example, α_(τ)*k*_ is the condition effect on the non-decision time and γ_(τ)_ reflects the change in non-decision time (seconds) due to a one SSVEP unit difference across two participants.

Subject-level drift rates δ_*jk*_, diffusion coefficients ς_*jk*_, and non-decision times τ_*jk*_ were assumed to be drawn from normal distributions with means of the form α_*k*_ + ***x*^⊺^_*j*_γ** where α_*k*_ is condition *k*'s effect on the subject-level cognitive parameter, ***x***_*j*_ is a vector of principal components, and **γ** is a vector of regression coefficients (i.e., the effect of each principal component on the cognitive parameter). The graphical representation of the model is provided in Figure 5. The priors of the variance parameters are the same as in **Model 2**. Weakly informative prior distributions of 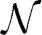
(0.0, 10) were given to the weight variables that make up the vectors **γ**_(δ)_, **γ**_(ς)_, and **γ**_(τ)_. The other hyperpriors and priors were:

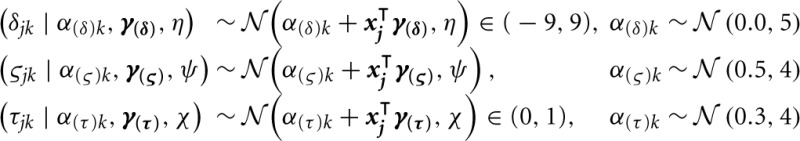



### 2.8. Posterior sampling

We used the JAGS software (Plummer, 2003) to analyze the data by drawing samples from the joint posterior distribution of the parameters of the hierarchical models. To compute the likelihood function associated with the assumed decision making process (the Wiener distribution), we used the *jags-wiener* module (Wabersich and Vandekerckhove, 2013). This allowed us to explain accuracy and response time distributions within conditions and across subjects. For each model, samples from the posterior distributions of the parameters were found by running JAGS with six Markov Chain Monte Carlo (MCMC) chains of length 21000, with 1000 burn-in (discarded) samples and a thinning parameter of 10 (keeping only every 10th sample) resulting in six joint posterior distribution estimates of 2000 samples each. We used the $\hat{R}$ statistic to compare within-chain variance to between-chain variance in order to assess convergence of the MCMC algorithm (Gelman and Rubin, 1992).

### 2.9. Posterior predictive distributions

To quantify model fit, in-sample posterior predictive distributions of accuracy-RTs from 5000 simulated experiments were estimated by sampling from the posterior distributions of subject-level parameters for each of the three models. That is, *s* = 1, …, 5000 samples were randomly drawn from the subject-level posterior distributions of the model parameters producing 5000 × 1 column vectors for each drift rate **δ**^(*s*)^_*jk*_, diffusion coefficient **ς**^(*s*)^_*jk*_, and non-decision time **τ**^(*s*)^_*jk*_. The samples $\left(\delta_{jk}^{\left(s\right)},\;\varsigma_{jk}^{\left(s\right)},\;\tau_{jk}^{\left(s\right)}\right)$ were used to generate accuracy-RT samples from the Wiener distribution [with the rejection sampling algorithm described in Tuerlinckx et al. (2001)].

In order to find candidate PLI predictors for **Model 3** and also to gauge the ability of each model type to predict new subjects' behavioral data, *in-sample* and *out-of-sample* posterior predictive distributions were generated using the PLI coefficients and posterior distributions of the *condition*-level parameters to find predictive distributions of the *subject*-level parameters. This procedure does not use samples from the subject-level posterior distributions directly, but estimates the subject-level parameters from the posteriors of the condition-level parameters and EEG covariates before finding a posterior predictive distribution of accuracy-RTs. Samples from the posterior predictive distribution of subject *j*'s mean drift rate on a trial in condition *k* are drawn from a normal distribution with mean **α**^(***s***)^_(**δ**)***k***_ + ***G***^(*s*)^_(**δ**)_***x*_*j*_** where ***x*_*j*_** is the vector of subject *j*'s principal component PLI values, **α**^(***s***)^_**(δ)*k***_ are samples from the posterior distribution of condition *k*'s effect on drift rate, and ***G*^(*s*)^_(**δ**)_** is a matrix consisting of samples from the posterior distributions of the PLI coefficients for drift rate. For in-sample prediction, we fit different possible forms of **Model 3**, with different numbers of principal components, 17 times each to generate in-sample posterior distributions to find candidate principal components. Then for out-of-sample prediction, we fit different possible forms of **Model 3**, with the resulting candidate principal components, 17 times with each participant removed from the data set. In the previously mentioned example, both the condition effect on drift rate and PLI coefficients are estimated from the model with all subjects except *j* for out-of-sample prediction.

## 3. Results

For all models and all parameters, convergence of the Monte Carlo chains was satisfactory: $\hat{R}$ ≤ 1.01 for all parameters ($\hat{R}$ ≥ 1.10 is conventionally taken as evidence for non-convergence; Gelman and Rubin, 1992).

### 3.1. Model 1: no individual differences

Marginal posterior distributions of the parameters of **Model 1** are plotted in the Supplemental Materials' Figure 8. The variability of evidence units gained per second ς_*k*_ increased as BR variance grew. Evidence units gained per second, drift rate δ_*k*_, was found to decrease both with larger contrast noise and larger BR. The parameter estimates seem to show a complex interaction effect of BR and contrast noise on non-decision time τ_*k*_. However, the results from **Model 2** will indicate that **Model 1** is sufficiently misspecified that this interaction cannot be interpreted in a meaningful way.

### 3.2. Model 2: individual differences

The marginal posterior distributions of the condition-level parameters are shown in Figure 8 of the Supplementary Materials. At the condition level, the effects of the experimental manipulations on drift rate and the diffusion coefficient remain similar to the results of **Model 1**: Mean drift rates ν_*k*_ were found to decrease as BR variance grew, smaller mean drift rates were observed in the high visual noise condition, and mean diffusion coefficients μ_*k*_ increased as BR variance grew. Main effects on the condition-level non-decision time not clearly observable in **Model 1** were found in **Model 2**. Mean non-decision time θ_*k*_ was slow when the BR variance was high, and participants were estimated to have quick non-decision times in low visual noise conditions.

The complex interactive pattern of non-decision times obtained in **Model 1** no longer appears.

By adding subject-level parameters, the current model not only provides a clearer picture of condition-level behavior of all participants, but describes the *individual differences* of the participants modeled by the subject-level parameters, δ_*jk*_, ς_*jk*_, and τ_*jk*_. Posterior distributions for the subject-level parameters of the easiest condition (±30° BR and 30% noise) are provided in the Supplemental Materials' Figure 9. Due to subject-level parameters deviating from the condition-level parameter's means, this model is able to predict within-sample data well-compared to the previous model. Percent variances explained (*R*^2^_pred_) of correct-RT subject medians by within-sample posterior prediction are provided in Table 1. **Model 2** explains at least 86.3% of median correct-RT between-subject variance in each condition.

**Table 1:** **Percentage of between-subject variance in correct-RT medians explained by in-sample and out-of-sample prediction (*R*^2^_*pred*_) for each experimental condition**.

**Rotation**	**Noise**	**In-sample prediction**			**Out-of-sample prediction**		
		**M1**	**M2**	**M3**	**M1**	**M2**	**M3**
±30°	30%	−0.1%	94.3%	95.0%	−13.5%	−11.8%	31.9%
±35°	30%	−0.1%	95.6%	95.8%	−12.3%	−11.7%	27.6%
±40°	30%	−0.5%	92.2%	92.1%	−12.5%	−11.5%	19.9%
±30°	45%	−1.2%	86.3%	87.4%	−15.1%	−11.7%	29.4%
±35°	45%	−0.2%	92.3%	91.6%	−12.0%	−13.6%	22.8%
±40°	45%	0.2%	92.6%	91.9%	−11.9%	−15.0%	28.0%
±30°	60%	−0.7%	93.1%	92.8%	−12.9%	−13.0%	18.6%
±35°	60%	−2.3%	92.5%	92.6%	−14.7%	−13.5%	13.3%
±40°	60%	−0.6%	90.9%	91.2%	−13.8%	−18.0%	26.2%

*The in-sample predictive ability of the no-individual differences **Model 1** was unsurprisingly poor, while the in-sample predictive ability of individual differences models (with and without EEG regressors, **Model 2** and **Model 3**, respectively) explained most of the variance of correct-RT subject medians. Out-of-sample prediction was performed by using an iterative leave-one-subject-out procedure, first by obtaining posterior distribution estimates for each parameter by modeling all but one participant's behavior and EEG data and then estimating the left-out participant's correct-RT distribution using the resulting model fit and the left-out participant's EEG. Models without EEG regressors (i.e., **Model 1** and **Model 2**) are poor choices for new participant behavior prediction. The model with a noise principal component and a signal principal component of the phase-locked EEG as covariates of diffusion model parameters (**Model 3**) more accurately predicts new participants' correct-RT behavior. Negative values indicate overdispersion of the model prediction (due to posterior uncertainty) relative to the real data*.

### 3.3. Model 3: individual differences with neural correlates

The results of **Model 2** clearly demonstrate differences between participants' cognition in the perceptual decision making task. We were further able to explain the differences in the cognitive variables using the neural data: **Model 3** was fit in a similar manner to **Model 2**, but additionally included principal components of the steady-state PLIs as regressors, as represented by the vector *x*_*j*_, on the subject-level model parameters.

We generated in-sample posterior predictive distributions using condition-level parameter posterior distributions (as opposed to in-sample posterior prediction from subject-level parameters), PLI coefficient posterior distributions, and PLI variables from each subject to find principal components that best predicted correct RT distributions. A plot of in-sample unexplained median correct-RT between-subject variance as a decreasing function of number of principal component (PC) regressors included in the model is provided in Figure 10 of the Supplemental Materials. Based on this analysis, PCs 2, 4, and 7 of both the noise and signal sets were tested furodel that best predicted out-of-sample correct-RT distributions by using noise component 2 and signal component 7 as exogenous PLI regressors other to find the model that best predicted out-of-sample RT of correct responses.

**Model 3** was the model that best predicted out-of-sample correct-RT distributions by using noise component 2 and signal component 7 as exogenous PLI regressors on the diffusion model parameters. It should be noted that the amount of variance of the original PLI data explained by each PC is not reflective of each PC's out-of-sample predictive power, just as the amount of variance of the original data explained by each PC is not reflective of its contribution to the model (Jolliffe, 1982). A table of percent between-subject variance of median correct-RT explained (*R*^2^_pred_) by out-of-sample prediction is provided in Table 1. Tables of percent between-subject variance of mean, 25th percentile, and 75th percentile correct-RT explained by out-of-sample prediction are provided in the Supplemental Materials. A new paricipant's correct-RT distribution in each condition can be more accurately predicted using the participant's EEG in **Model 3**'s framework than by using **Model 1**'s or **Model 2**'s framework. 31.9% of the between-subject variance of the easiest condition's median correct-RT is explained by out-of-sample prediction.

To aid in interpretation, the posterior distributions of the regression coefficients for each PC were projected into the PLI coefficient space by multiplying the matrix of PC coefficient posterior samples **G** by the inverse-weight matrix **V** from the PCA algorithm which projects the PCs into the PLI data space. The result **GV** are samples from the posterior distributions of the regression coefficients for each PLI variable. This transformation was performed once for each of the noise and signal variable sets.

**Figure 6:**
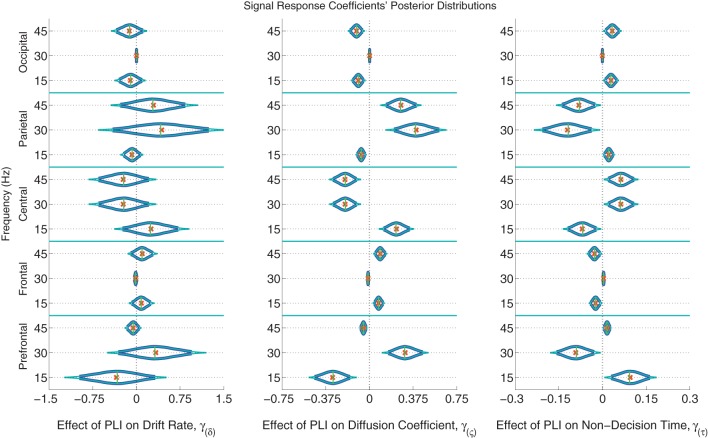
**The marginal posterior distributions of the signal PLI coefficients**. I.e., the effects of signal enhancement, as measured by a steady-state phase-locking index (PLI), on the evidence accumulation rate (drift rate; in evidence units per second), variance in the evidence accumulation process (the diffusion coefficient; in evidence units per second), and non-decision time during the response interval (in seconds). Dark blue posterior density lines indicate 95% credible intervals while smaller teal lines indicate 99% credible intervals. Small horizontal green lines embedded in density curves indicate the median of the posterior distributions while the orange crosses indicate posterior means. There is an effect of signal response on the diffusion coefficient and non-decision time that is complex across frequencies and scalp location. A participant whose PLI responses at all locations and frequencies are 0.2 units greater than another participant's responses is expected to have 0.061 evidence units per second larger evidence accumulation variances (where α = 1 evidence unit is required to make a decision) and have 18 ms faster non-decision times, leading to faster but less accurate responses. There was no evidence of an effect of attention to the signal on evidence accumulation rate (the drift rate).

The posterior distributions of the signal PLI coefficients are provided in Figure 6 with means, medians and 95% and 99% credible intervals. From the PC coefficient and PLI coefficient posteriors, it was clear that there is a complex signal response at multiple frequencies and cortical locations on the diffusion coefficient and non-decision time. Participants with larger signal occipital 15 and 45 Hz PLIs are expected to have smaller variances in the evidence accumulation process (diffusion coefficients) than those participants with smaller occipital signal PLIs. However, the opposite effect is found in the frontal electrodes with large 15 and 45 Hz PLIs being associated with larger evidence accumulation variances. Larger signal responses at 30 and 45 Hz in parietal electrodes is also associated with larger diffusion coefficients. The effect of signal response on non-decision time is also complex but closely related to the effect of signal response on the diffusion coefficient. No evidence of an association between participants' differences in signal response to differences in evidence accumulation rates (drift rates) was found.

**Figure 7:**
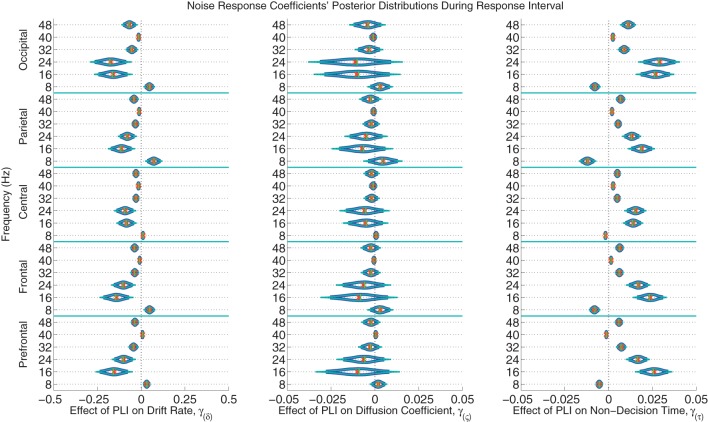
**The marginal posterior distributions of the noise PLI coefficients**. I.e., the effects of noise suppression, as measured by a steady-state phase-locking index (PLI), on the evidence accumulation rate (drift rate; in evidence units per second), variance in the evidence accumulation process (the diffusion coefficient; in evidence units per second), and non-decision time (in seconds) during the response interval. Dark blue lines indicate 95% credible intervals, smaller teal lines indicate 99% credible intervals, horizontal green lines indicate posterior medians, and the orange exes indicate posterior means. At noise harmonic frequencies (16, 24, 32, and 48 Hz) during the response interval, those subjects who suppressed noise had faster evidence accumulation rates; this effect was found at all electrode groups. However, noise enhancement at 8 Hz was associated with slower evidence accumulation. Furthermore, those subjects who better suppressed noise at the same harmonic frequencies had faster non-decision times. For example, a participant whose PLI responses were suppressed 0.2 units more than another participant's responses at all locations and frequencies during the response interval is expected to accumulate 0.288 evidence units per second faster (where α = 1 evidence unit is required to make a decision) and have 48 ms faster non-decision times, leading to faster and more correct responses. There was no evidence of an effect of attention to the visual noise on variance in evidence accumulation (the diffusion coefficient).

The posterior distributions of the noise PLI coefficients from the response interval are provided in Figure 7. The posterior distributions of the noise PLI coefficients from the noise interval are provided in the Supplemental Materials' Figure 11. In all noise harmonic frequencies during the noise interval and most harmonic frequencies (16, 24, 32, and 48 Hz) during the response interval, those subjects who had smaller PLIs at all electrode locations had faster evidence accumulation rates (drift rates). This finding suggests that those subjects who better suppressed the stimulus noise accumulated correct evidence faster. Furthermore, a similar effect was found on non-decision time. Noise suppression in the harmonic frequencies was associated with smaller non-decision times across subjects. However, smaller PLIs at 8 Hz were associated with slower evidence accumulation and faster non-decision times. Looking at these effects as a whole, those subjects with more suppressed responses to the noise at all frequencies had larger drift rates and smaller non-decision times leading to faster, more accurate responses. As a plausible but oversimplified example, a participant whose PLI responses at all frequencies and locations was suppressed 0.2 units more than another participant during both the noise and response intervals is expected to accumulate 0.418 evidence units per second faster than another participant and have a 70 ms faster non-decision time. There was little to no evidence of an effect of individual variation in brain responses to noise on within-trial evidence accumulation variability (the diffusion coefficient).

## 4. Discussion

We have shown that a Bayesian diffusion model framework with hierarchical participant-level parameters is useful in describing individual differences in the rate of evidence accumulation, variance in evidence accumulation process, and preprocessing and/or motor response time in a novel perceptual decision making paradigm. Assuming the model describes the relationship between cognition and behavior sufficiently well, we are able to infer cognitive differences among participants. Furthermore, we have shown that differences in participants' attention as measured by SSVEPs relate to some of these differences in participants' cognition.

Individual differences in the rates of evidence accumulation (drift rates) were partially explained by individual differences in noise suppression as measured by SSVEPs. Participants who better suppressed noise at high frequencies during the both the preparatory period (noise interval) and the decision period (response interval) were able to accumulate correct evidence faster, which led to more accurate, faster response times. Furthermore, those individuals who better suppressed noise in the same frequency bands and locations had faster non-decision times (preprocessing and/or motor response speed). This effect on non-decision time is hypothesized to be reflective of faster preprocessing time in subjects who better suppressed noise since we do not expect noise suppression to affect motor response speed. Both findings suggest a role of noise suppression in beta and gamma EEG frequency bands on the speed of evidence accumulation and preprocessing prior to evidence accumulation in perceptual decision making tasks.

Enhancement of signal was found to describe individual variation in “randomness” of evidence accumulation within trials (as measured by the diffusion coefficient). Participants who did not properly enhance signal in occipital, central, and pre-frontal electrodes had the most variable evidence accumulation processes. There is also evidence that a participant's enhancement of signal may have affected their preprocessing time in a complex way across frequencies and cortical locations. This suggests that signal enhancement in beta and gamma EEG frequency bands affect within-trial evidence accumulation variance and preprocessing in perceptual decision making.

In summary, from the results of the modeling procedure it was found that some individual variation in evidence accumulation speed (drift rate) is explained by noise suppression, some individual variation in evidence accumulation variance (diffusion coefficient) is explained by signal enhancement, and some individual variation in non-decision time (presumably preprocessing time) is explained by both noise suppression and signal enhancement.

The usefulness of the model with SSVEP attention measures as regressors is not only in its descriptive ability, but also in its predictive ability. New subject correct-RT behavior was not accurately described by the model without individual differences nor the model with individual differences. But by explicitly including individual differences with neural covariates in hierarchical models, the correct RT distributions of new subjects with known neural measures are more accurately predicted. We expect the addition of the phase-locking index of SSVEPs to be predictive of behavior in any perceptual decision making paradigm, especially if used in a hierarchical Bayesian framework. Theoretically the hierarchical EEG-diffusion model will also be able to predict the PLI measures of a missing participant given a participant's behavioral data. We will explore the practicality of such predictions in future studies. Possible applications of behavioral and neural data prediction include: (a) the ability to interpolate data from incomplete behavioral data sets (b) the ability to interpolate data from incomplete neural data sets (c) more powerful statistical inference through simultaneous accounting for changes in behavior and neural data.

In the future for both hypothesis testing and response-RT prediction, latent variables linearly or non-linearly related to the EEG covariates can be included with the cognitive model in a hierarchical Bayesian framework (see Vandekerckhove, 2014, for details]. The benefits of such an analysis would be: to choose neural covariates maximally descriptive or predictive of the data, choose electrodes and frequencies maximally descriptive or predictive of the data, reduce the number of covariates, and reduce the multicollinearity of the covariates by assuming there exist underlying variables related to multiple EEG covariates. In the present study, the problems of multicollinearity and variable overabundance were overcome with two principal component analyses (PCAs). PCAs do not extract mixtures of the data which are most descriptive or predictive of the model parameters but instead extract mixtures of the data which are uncorrelated. A shortcoming of this study is that we did not pick frequencies and cortical locations that were maximally predictive of behavior as exogenous variables. Cortical locations naively based upon large non-focal groupings were chosen. Instead of performing a non-Bayesian PCA before submitting the neural data to the Bayesian algorithm, a linear mixture of neural data that best describes the cognitive model parameters could be extracted from the Bayesian algorithm itself, analogous to a partial least squares regression in a non-Bayesian approach (see Krishnan et al., 2013, for an example). In order to use this latent variable technique, the model must be run on a training set using a subset of the EEG data and then run on a test set to measure out-of-sample model predictive ability. This would result in a data reduction of the EEG that best predicts behavior in the context of the model.

## Funding

Ramesh Srinivasan and Michael D. Nunez were supported by NIH grant 2R01MH68004. Michael D. Nunez's support was supplemented by the John I. Yellott Scholar Award from the Cognitive Sciences Dept., University of California, Irvine. Joachim Vandekerckhove was supported by NSF grant #1230118 from the Methods, Measurements, and Statistics panel and grant #48192 from the John Templeton Foundation.

### Conflict of interest statement

The authors declare that the research was conducted in the absence of any commercial or financial relationships that could be construed as a potential conflict of interest.
