# Supplementary material for: Individual differences in attention influence perceptual decision making
Source: Front Psychol. 2015 Feb 5;8:18. doi: 10.3389/fpsyg.2015.00018 (PMC4329506; doi:10.3389/fpsyg.2015.00018)
Supplement: Supplementary file 1 [file Presentation1.PDF]

Frontiers in Psychology

Research Article
8 December 2014

# Supplementary Material: Individual differences in attention influence perceptual decision making

**Michael D. Nunez** [1,*], **Ramesh Srinivasan** [1,2] **and Joachim Vandekerckhove** [1,3]

[1] *Department of Cognitive Sciences, University of California—Irvine, Irvine, CA, USA*
[2] *Department of Biomedical Engineering, University of California—Irvine, Irvine, CA, USA*
[3] *Institute for Mathematical Behavioral Sciences, University of California—Irvine, Irvine, CA, USA*

Correspondence*:
Mr. Michael D. Nunez
Department of Cognitive Sciences, University of California, Irvine, USA,
mdnunez1@uci.edu

**Modeling individual differences in perceptual decision making**

## 5 SUPPLEMENTARY EQUATIONS

### 5.1 PREDICTIVE POWER AS MEASURED BY $R^2_{\text{PRED}}$

We define $R^2_{\text{pred}}$ as a measure of the percentage of total between-subject variance of a statistic $T$ (e.g. the correct-RT median) explained by in-sample or out-of-sample prediction. It is a function of the mean squared error of prediction (MSEP) and the sample variance of the statistic $T$ based on a sample size of $n = 17$ subjects. $R^2_{\text{pred}}$ is defined in Equation 3.

$$R^2_{\text{pred}} = 1 - \frac{\sum_{j=1}^{17}(T_j - T_{(\text{pred})j})^2/16}{\sum_{j=1}^{17}(T_j - \bar{T})^2/16} = 1 - \frac{\text{MSEP}_T}{\widehat{\text{Var}[T]}} \qquad (3)$$

### 5.2 SUPPLEMENTARY FIGURES AND TABLES

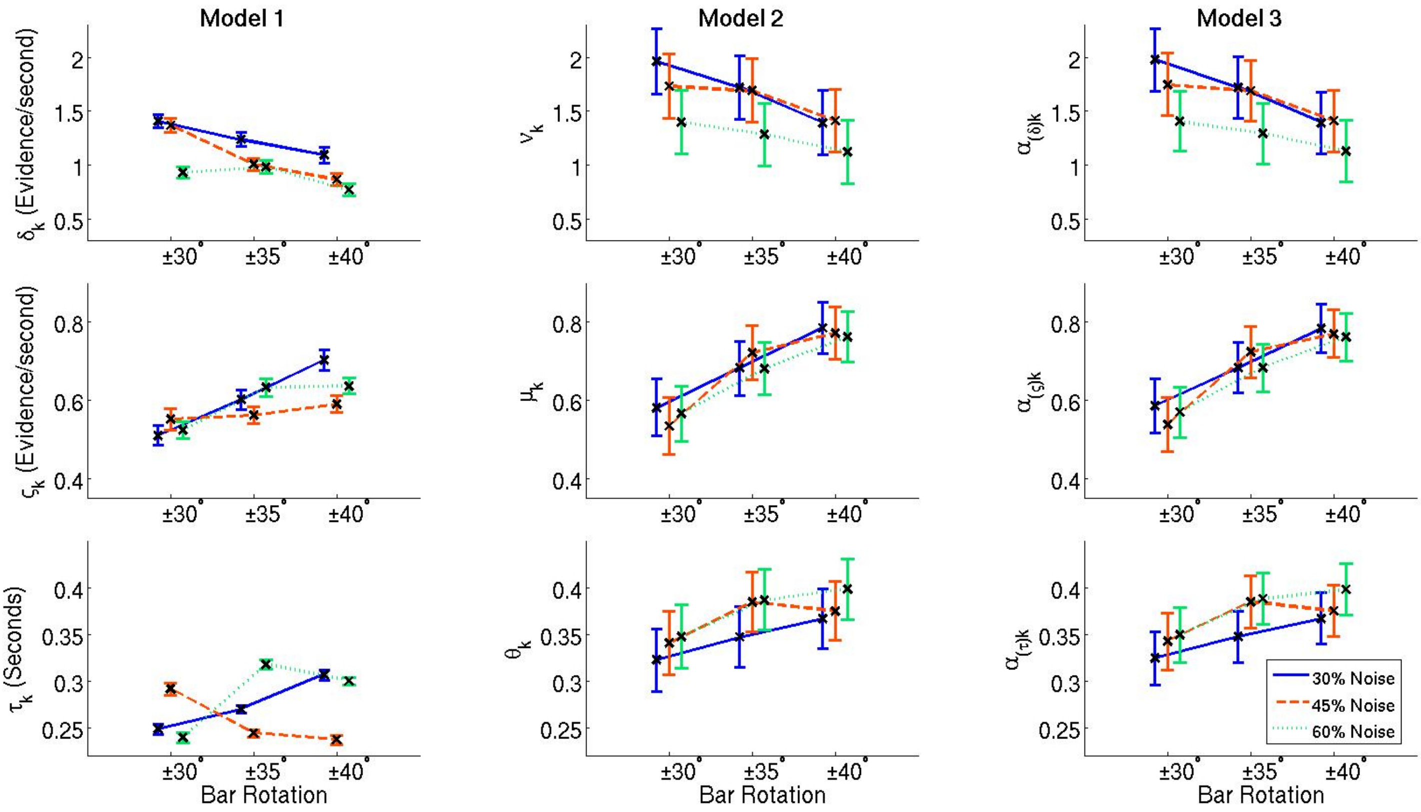


**Figure 8.** The posterior means as represented by the black crosses and 95% credible intervals of the condition level parameter posterior distributions. From top to bottom, the rows correspond to the condition level drift rate, diffusion coefficient, and non-decision time posterior distributions for each model. The left column displays posterior samples of the parameters in **Model 1**, the model without assumed individual differences. The middle column shows condition level posterior distributions from **Model 2**. The larger variances in the posterior samples are due to explicitly modeled individual differences. The right column shows the credible intervals of the condition effects on each variable of **Model 3**. As expected the model with exogenous neural data as predictors of the subject level parameters has no effect on the condition level parameters.

| Correct-RT Mean | Out-of-sample Prediction | | |
|---|---|---|---|
| Condition | M1 | M2 | M3 |
| ±30° BR 30% Noise | −25.2% | −23.2% | −4.0% |
| ±35° BR 30% Noise | −38.1% | −21.8% | 6.5% |
| ±40° BR 30% Noise | −26.3% | −17.1% | 18.9% |
| ±30° BR 45% Noise | −21.4% | −23.0% | −0.8% |
| ±35° BR 45% Noise | −43.1% | −18.1% | 16.0% |
| ±40° BR 45% Noise | −73.8% | −17.0% | 25.3% |
| ±30° BR 60% Noise | −47.5% | −28.6% | −3.3% |
| ±35° BR 60% Noise | −44.0% | −29.4% | −11.7% |
| ±40° BR 60% Noise | −48.8% | −13.6% | 36.8% |

**Table 2.** Percentage of between-subject variance in correct-RT means explained by out-of-sample prediction ($R^2_{pred}$) for each experimental condition.

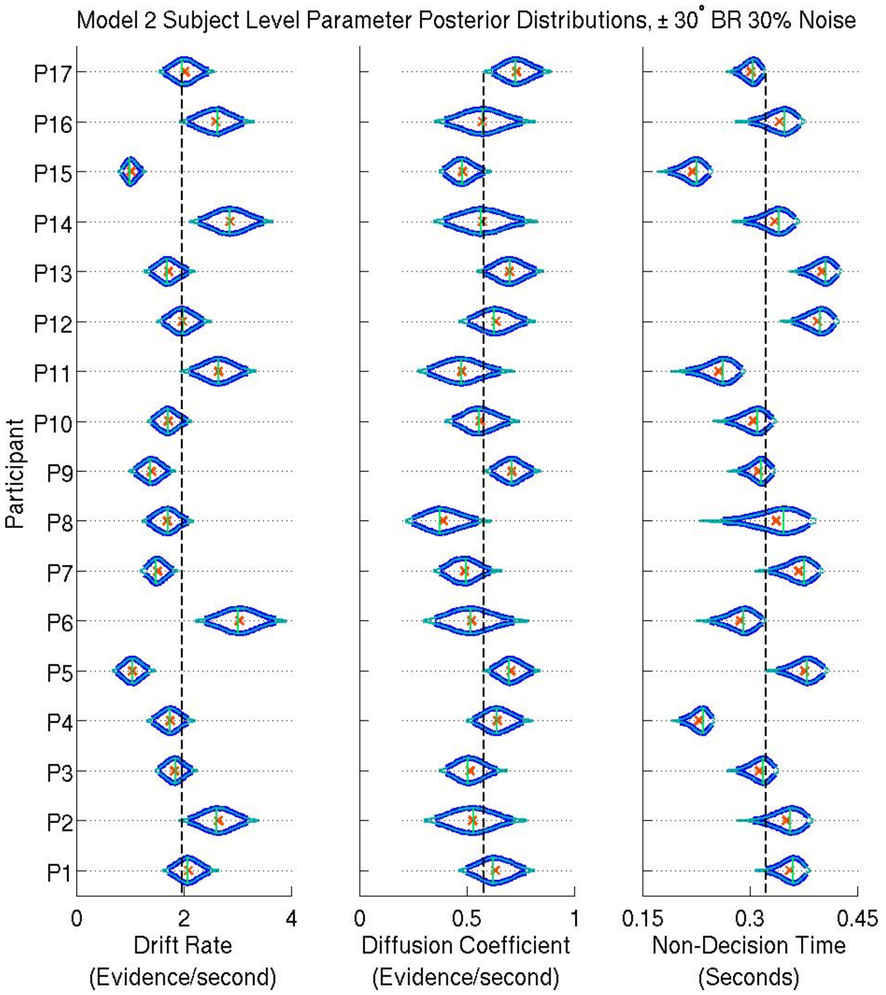


**Figure 9.** Posterior distributions for the subject level parameters of the $\pm 30°$ bar rotation and 30% noise condition. The thick blue line of the posterior density indicates 95% coverage, the density between the 2.5th and 97.5th percentiles. The thin teal line of the posterior density indicates 99% coverage, the density between the .5th and 99.5th percentiles. The vertical black line is the mean value of the condition level parameters. Nine subject level drift rates $\delta_{j1}$ deviate significantly at the 95% level from the mean posterior condition level drift rate $\nu_k$. Three subject level diffusion coefficients $\varsigma_{j1}$ deviate significantly at the 95% level from the mean posterior condition level diffusion coefficient $\mu_k$. And nine subject level non-decision times $\tau_{j1}$ deviate significantly at the 95% level from the mean posterior condition level non-decision time $\theta_k$. It is clear from these results that there were differences between participants' cognition in the easiest condition.

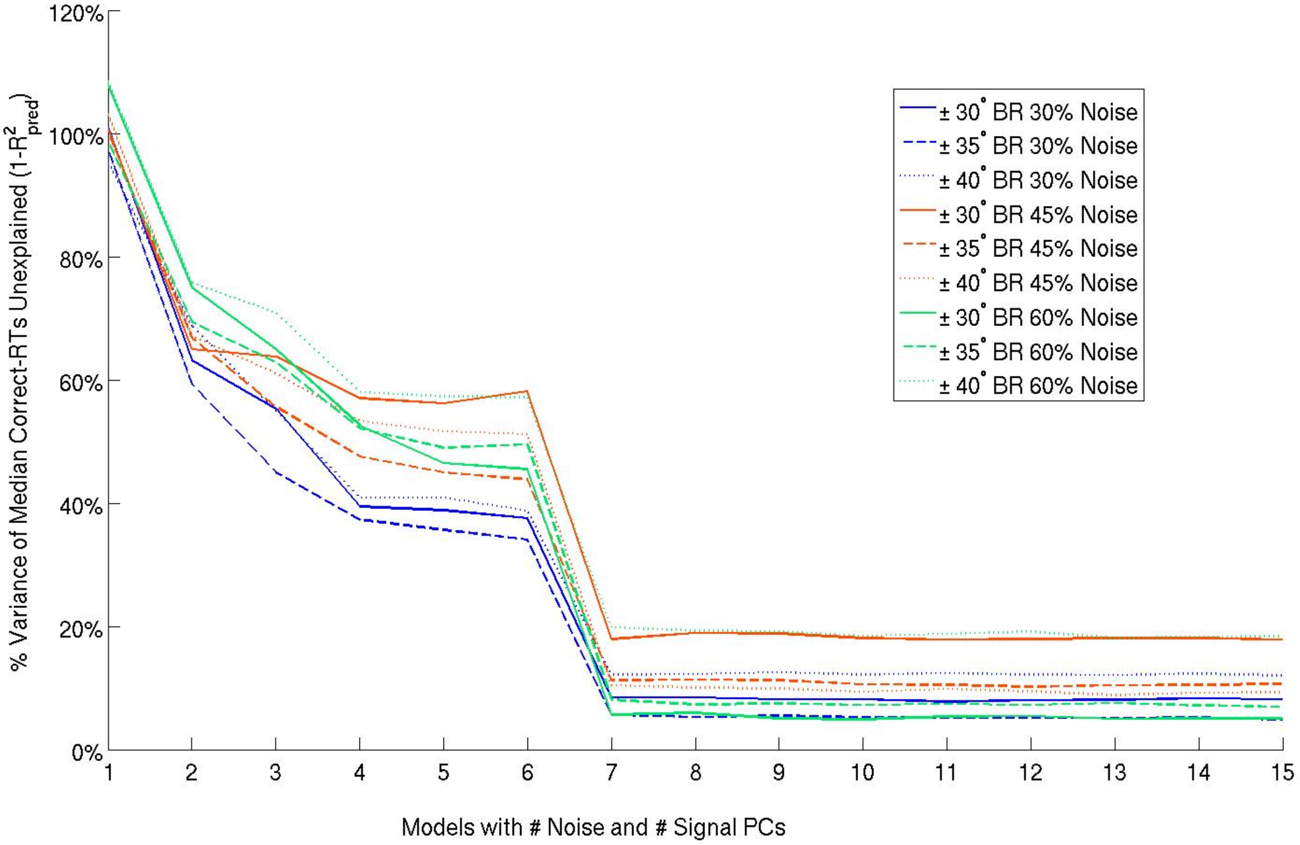


**Figure 10.** Two principal components analyses (PCAs) on the noise and signal PLI variables were performed to obtain **Model 3**'s regressors. We generated in-sample posterior predictive distributions using samples from condition level parameter posterior distributions and EEG variables (to find subject level parameter predictive distributions) in order to find principal components that best predicted correct-RT distributions. Predictive power was measured as $R^2_{\text{pred}}$ of the subject correct-RT medians of each condition. Each model type is plotted versus $1 - R^2_{\text{pred}}$ which is a measure of the percent variance of a statistic (e.g. median correct-RT) unexplained by *in-sample* prediction. We iteratively added one principal component per variable set (signal variables and noise variables) to the model. Based on this analysis principal components 2, 4, and 7 of both the noise and signal sets were tested further to find the model that best predicted *out-of-sample* reaction time of correct responses.

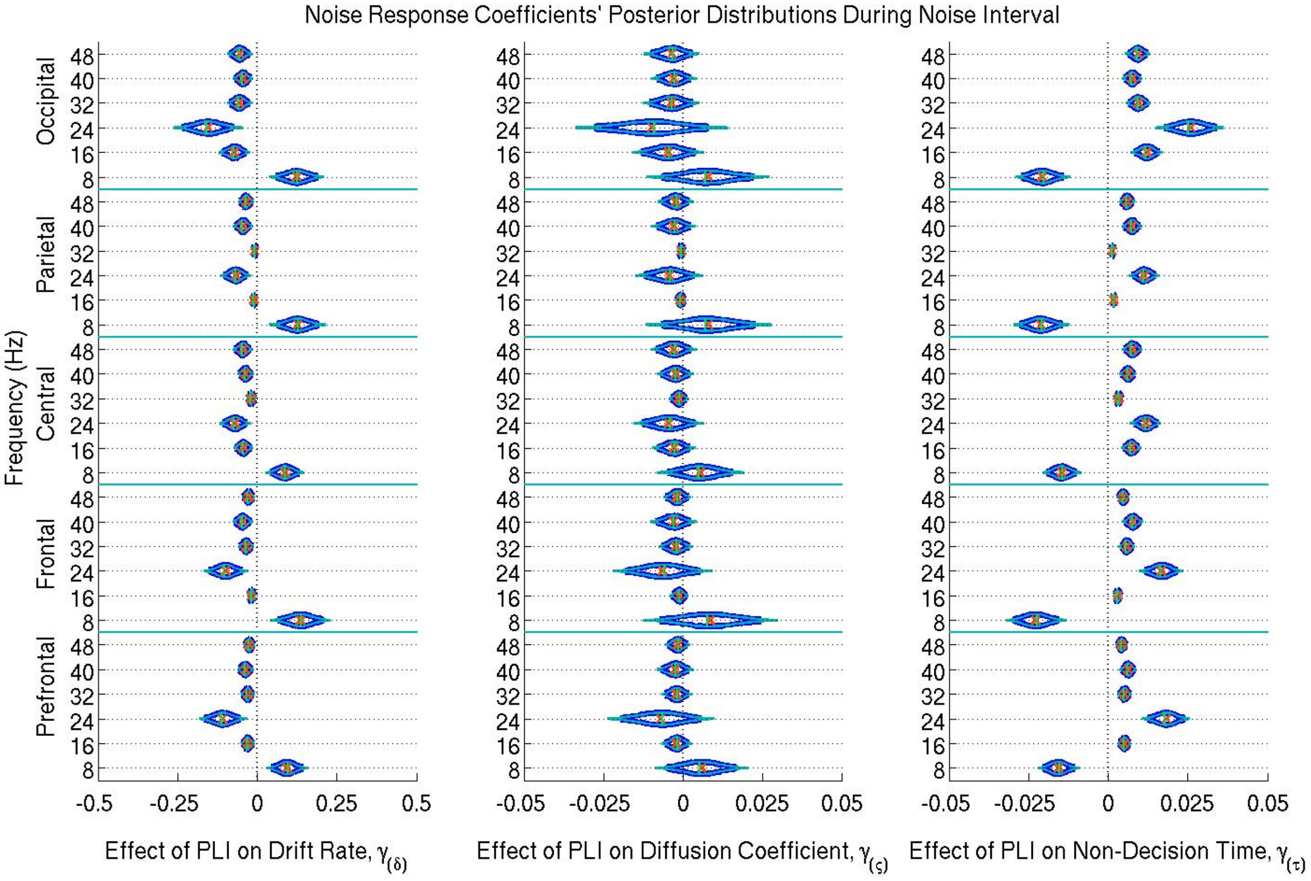


**Figure 11.** The marginal posterior distributions of the noise PLI coefficients on the drift rate, diffusion coefficient, and non-decision time during the noise interval. Dark blue lines indicate 95% credible intervals, smaller teal lines indicate 99% credible intervals, horizontal green lines indicate posterior medians, and the orange exes indicate posterior means. We consider noise PLI coefficients to be significant if the 95% credible intervals do not include 0. At all noise harmonic frequencies (16, 24, 32, 40 and 48 Hz) during the noise interval, those subjects who suppressed noise had faster evidence accumulation rates (drift rates); this effect was found at all electrode groups. However noise enhancement at 8 Hz was associated with slower evidence accumulation. Those subjects who better suppressed noise at harmonic frequencies also had faster non-decision times. E.g., a participant whose PLI responses were suppressed .2 units more than another participant's responses at all locations and frequencies during the noise interval is expected to accumulate 0.131 evidence units per second faster and have 22 ms faster non-decision times, leading to faster and more correct responses.

| Correct-RT 25th Percentile | Out-of-sample Prediction | | |
|---|---|---|---|
| Condition | M1 | M2 | M3 |
| ±30° BR 30% Noise | −50.2% | −24.0% | 23.6% |
| ±35° BR 30% Noise | −70.6% | −31.5% | 13.0% |
| ±40° BR 30% Noise | −51.8% | −26.5% | 15.8% |
| ±30° BR 45% Noise | −47.4% | −25.4% | 19.2% |
| ±35° BR 45% Noise | −97.6% | −30.9% | 16.6% |
| ±40° BR 45% Noise | −120.3% | −43.5% | −0.1% |
| ±30° BR 60% Noise | −110.2% | −49.0% | −5.3% |
| ±35° BR 60% Noise | −70.8% | −35.6% | 7.3% |
| ±40° BR 60% Noise | −79.2% | −45.7% | 1.7% |

**Table 3.** Percentage of between-subject variance in correct-RT 25th percentiles explained by out-of-sample prediction ($R^2_{pred}$) for each experimental condition.

| Correct-RT 75th Percentile | Out-of-sample Prediction | | |
|---|---|---|---|
| Condition | M1 | M2 | M3 |
| ±30° BR 30% Noise | −62.5% | −41.3% | −15.7% |
| ±35° BR 30% Noise | −61.3% | −21.8% | 0.4% |
| ±40° BR 30% Noise | −50.0% | −22.9% | 9.9% |
| ±30° BR 45% Noise | −39.1% | −27.3% | −20.7% |
| ±35° BR 45% Noise | −88.2% | −18.4% | 12.4% |
| ±40° BR 45% Noise | −193.2% | −24.0% | 13.2% |
| ±30° BR 60% Noise | −86.3% | −24.8% | −6.5% |
| ±35° BR 60% Noise | −82.4% | −33.6% | −19.5% |
| ±40° BR 60% Noise | −71.6% | −11.8% | 27.8% |

**Table 4.** Percentage of between-subject variance in correct-RT 75th percentiles explained by out-of-sample prediction ($R^2_{pred}$) for each experimental condition.
